# Combining the External Fixation and Microsurgical Osteoseptocutaneous Flap Transplantation Methods for Limb Salvage

**Published:** 2012-03-22

**Authors:** Musa Mateev, Chenyu Huang, Arstan Imanaliev, Shimpei Ono, Hiko Hyakusoku, Rei Ogawa

**Affiliations:** ^a^Department of Plastic and Reconstructive Microsurgery and Hand Surgery, National Hospital of Kyrgyzstan, Bishkek, Kyrgyzstan; ^b^Department of Plastic Surgery, Meitan General Hospital, Beijing, China; ^c^Department of Plastic, Reconstructive and Aesthetic Surgery, Nippon Medical School, Tokyo, Japan

## Abstract

**Background:** The correction of large area limb defects that are the result of congenital abnormalities, traumatic injury, inflammation, or tumors is a challenging task for clinicians. The need to restore the physical, mechanical, and cosmetic aspects of the limb results in a difficult balancing act between deformity repair and tissue reconstruction, between the soft tissue and bone reconstructions, and between the physical, mechanical, and esthetic restorations. **Methods:** Between 2003 and 2011, 59 patients with large area limb defects underwent 1- or 2-stage reconstructions that combined external fixation with microsurgical osteocutaneous flap transfer. In 1-stage reconstruction, the Ilizarov device was applied as a dynamic fixator before pathological bone resection. Free osteoseptocutaneous flap transplantation was then performed. This is suitable for simple bone defects with temporary continuity such as osteomyelitis. In 2-stage reconstruction, the Ilizarov device served as both an external fixator and tractor before debridement. This was followed by secondary free osteoseptocutaneous flap transplantation. This is suitable for complex bone defects like those seen in pseudarthrosis, fractures, and tumors. In all cases, the Ilizarov device was kept in position until bone union was confirmed by both surgeons and the radiologist. **Results:** All patients survived the procedure. Using Paley's classification system, there were 3 (5.1%) true complications, 2 (3.4%) obstacles, and 5 (8.5%) problems. **Conclusions:** The staged methods allowed the seamless repair and reconstruction of bone and combined it with soft tissue reconstruction. This simultaneously restored the limb function and esthetics with minimal costs in terms of time, money, and patient pain.

Complex large area tissue defects of the limbs that are the result of congenital abnormalities, traumatic injuries, inflammation, or tumors are often perceived as significant challenges by both orthopedic and plastic surgeons. Not only physical but also mechanical and cosmetic restoration is the goal and this should ideally be achieved with minimal costs in terms of time, money, and patient pain. Because the alignment and continuity of the bone has to be maintained while an adequate length and volume of bone and its overlying soft tissues is provided, it has been recommended that such surgery be conducted under the concerted efforts of both orthopedic and plastic surgeons.^1^

To reduce the complexity of limb salvage operations in daily practice, we proposed and tested the possibility of combining the methods of external fixation and free osteoseptocutaneous flap transplantation. This combined approach can be applied in 1-stage or 2-stage procedures depending on the clinical situation. In this approach, the Ilizarov device is applied as an external tractor and/or fixator. In 1-stage reconstruction, the Ilizarov device is applied as a dynamic fixator before pathological bone resection and primary free flap transplantation. This is suitable for simple bone defects with temporary continuity such as osteomyelitis. In 2-stage reconstruction, the Ilizarov device serves as both an external fixator and tractor before debridement. This is then followed by secondary free osteoseptocutaneous flap transplantation. This is suitable for complex bone defects such as those as seen in pseudarthrosis, fractures, and tumors. This approach means that the bone is repaired and reconstructed at the same time that the soft tissues are reconstructed. As a result, the functions and esthetics of the defective limb are restored simultaneously.

## PATIENTS AND METHODS

### Patients

Between 2003 and 2011, 59 patients underwent limb salvage operations. Patient age and sex, the cause of the scar, and the preoperative and postoperative sizes of the scar were recorded. No comorbidities such as diabetes were present in any of the patients.

### Application of the Ilizarov device

#### One-stage reconstruction

The monofocal Ilizarov device was applied before bone reconstruction to keep the shape and size of the affected bone equal to those of the contralateral side bone. Full resection of the pathological bone was then performed and the resulting defect was repaired by a standard microsurgical osteoseptocutaneous fibula transplantation from the contralateral side. To ensure the safety of the free flap, rehabilitation of the affected limb and donor leg was only allowed from the seventh day after surgery. X-rays were taken before surgery and every 2 weeks after surgery. The Ilizarov device was kept in position until bone union was confirmed by both surgeons and the radiologist.

**Two-stage reconstruction**

The Ilizarov device was applied to eliminate and repair deformities in cases with congenital pseudarthrosis, complex fractures, and bone tumors.

**Stage 1**

The monofocal Ilizarov device was applied to correct the axial, angular, and translational deformities of the affected bones. Extremity traction was also continued after surgery by turning the nuts on the treaded rods at an average speed of 1 mm per day (0.25 mm/time × 4 times/day); this served to allow the length to become similar to that of the bone on the contralateral side.

**Stage 2**

Microsurgical osteoseptocutaneous fibula transplantation was performed. To ensure the safety of the free flap, rehabilitation of the affected limb and donor leg was only allowed from the seventh day after surgery. X-rays were taken before surgery and every 2 weeks after surgery. The Ilizarov device was kept in position until bone union was confirmed by both surgeons and the radiologist. The principles outlined in the Declaration of Helsinki have been followed.

## RESULTS

The average age of the patients was 23 years, and 15.3% were female and 84.7% were male. All patients survived the staged reconstructions consisting of the Ilizarov and free flap transplantation steps.

Postoperative complications were defined according to Paley's classification system. Thus, *problems* were defined as difficulties that did not require operative intervention to be resolved; *obstacles* referred to difficulties that did require operative intervention; and true *complication*s indicated intraoperative injuries where all problems were not resolved by the end of treatment.^2^ The postoperative complications of our staged reconstructions are listed in Table 1. Of the 59 cases, there were 3 (5.1%) true complications, 2 (3.4%) obstacles, and 5 (8.5%) problems. Of these, total necrosis of the fibular transplant due to arterial thrombosis associated with vascular anastomosis occurred in 2 cases of traumatic tibial defects. Venous thrombosis associated with vascular anastomosis was seen in one case of traumatic tibial defect; a reanastomosis was made on the saphenous vein the day after the primary anastomosis, with the result that the flap survived completely. Infection problems occurred in 2 cases of osteomyelitis after fibula transplantation and were resolved by draining and irrigation. Failure of union in the proximal part of the tibia occurred in 2 cases of congenital pseudarthrosis. One case was successfully treated by again applying the Ilizarov device to the proximal part; the other case had to undergo amputation. Stress-induced fracture of the fibula occurred in 3 cases 8 months after microsurgery; to repair this, the Ilizarov device was applied again. Complete consolidation was achieved in all cases.

### Typical cases

**Case 1: Congenital pseudarthrosis forearm**

**Figure 1:**
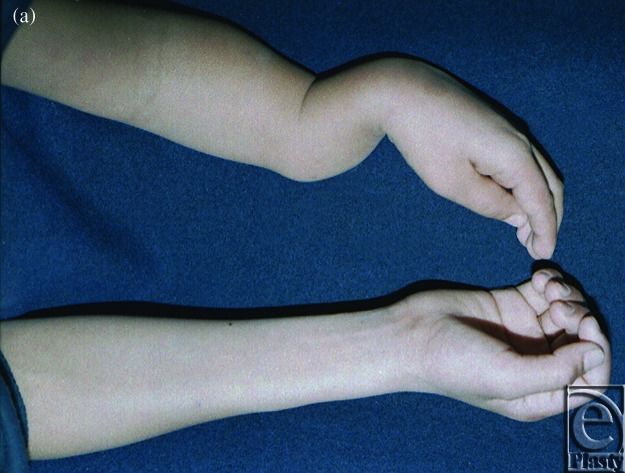

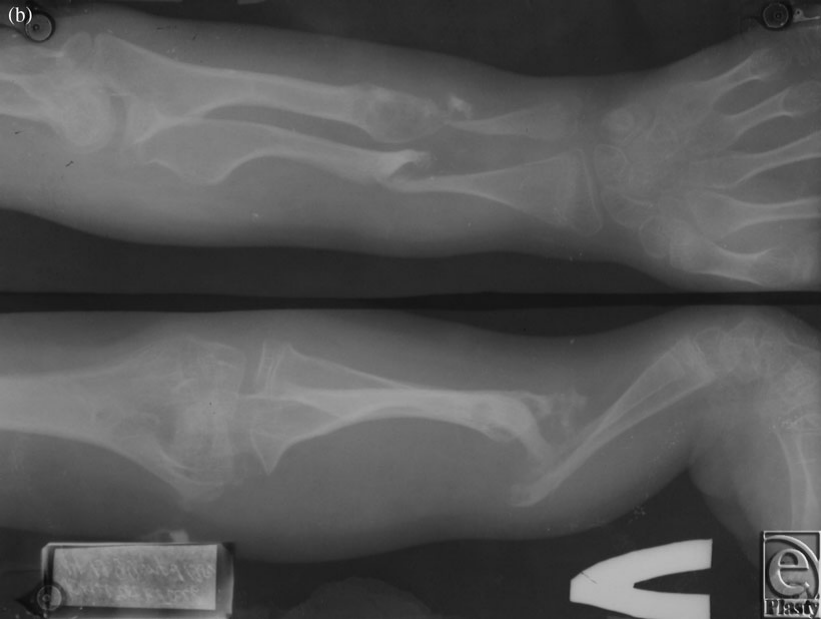

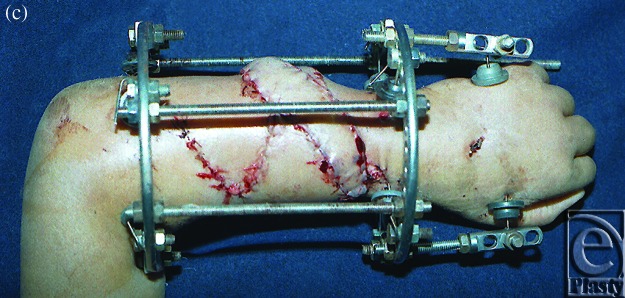

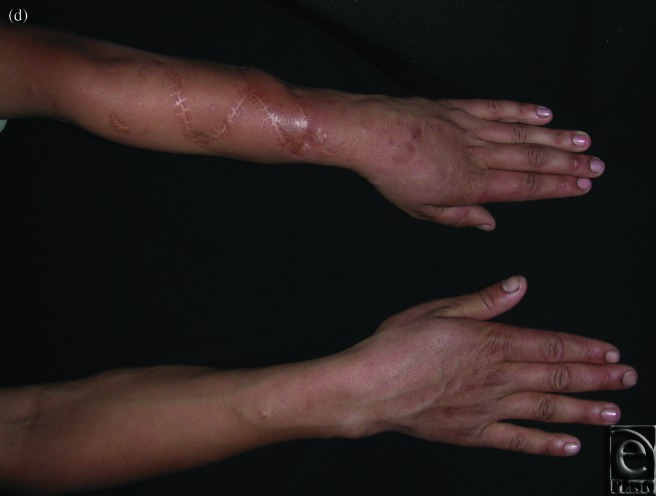

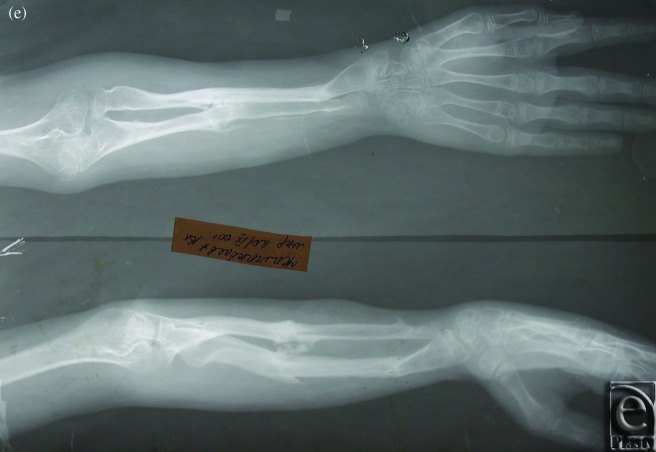
Case 1: Congenital pseudarthrosis forearm. (*a*) Preoperative view, (*b*) Preoperative x-ray, (*c*) External fixation, (*d*) The 1-year postoperative view, and (e) A 1-year postoperative x-ray.

The patient was an 8-year-old boy who suffered from congenital pseudarthrosis forearm type II on both the radius and the ulna. After resection and Ilizarov external fixation/traction, the discrepant 8-cm-long space between the 2 sides was repaired. A secondary free fibula osteoseptocutaneous flap was then harvested from the contralateral side and transferred to the affected side to cover the tissue defects (Figs 1a-1e).

**Case 2: Trauma of the lower leg**

**Figure 2:**
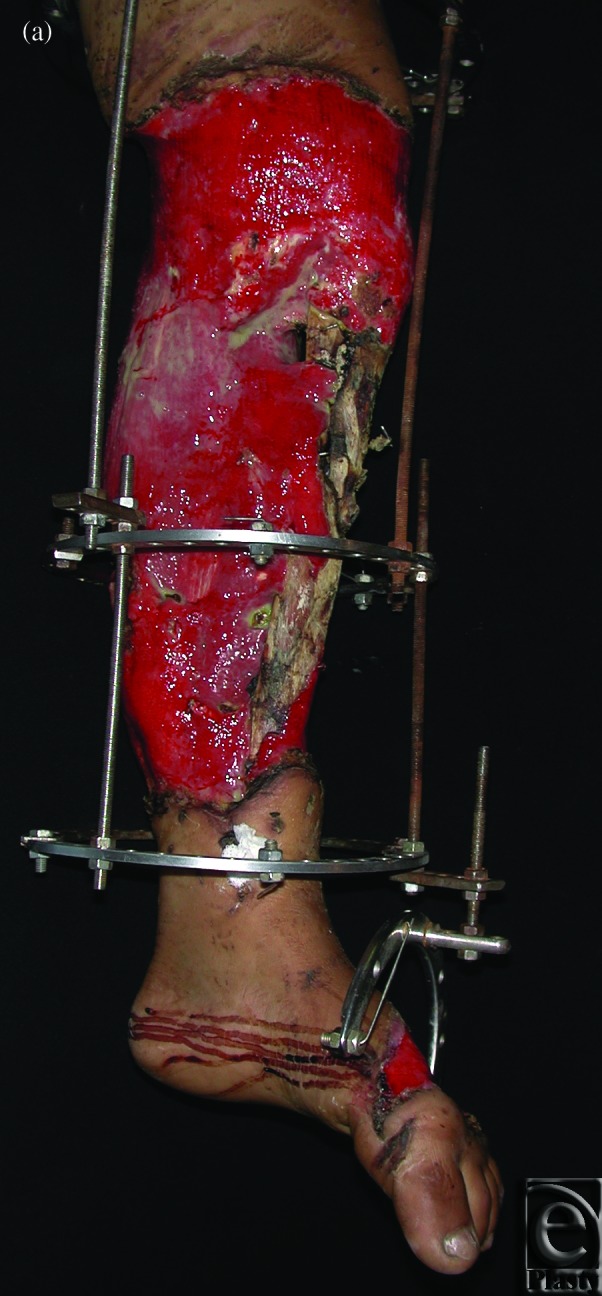

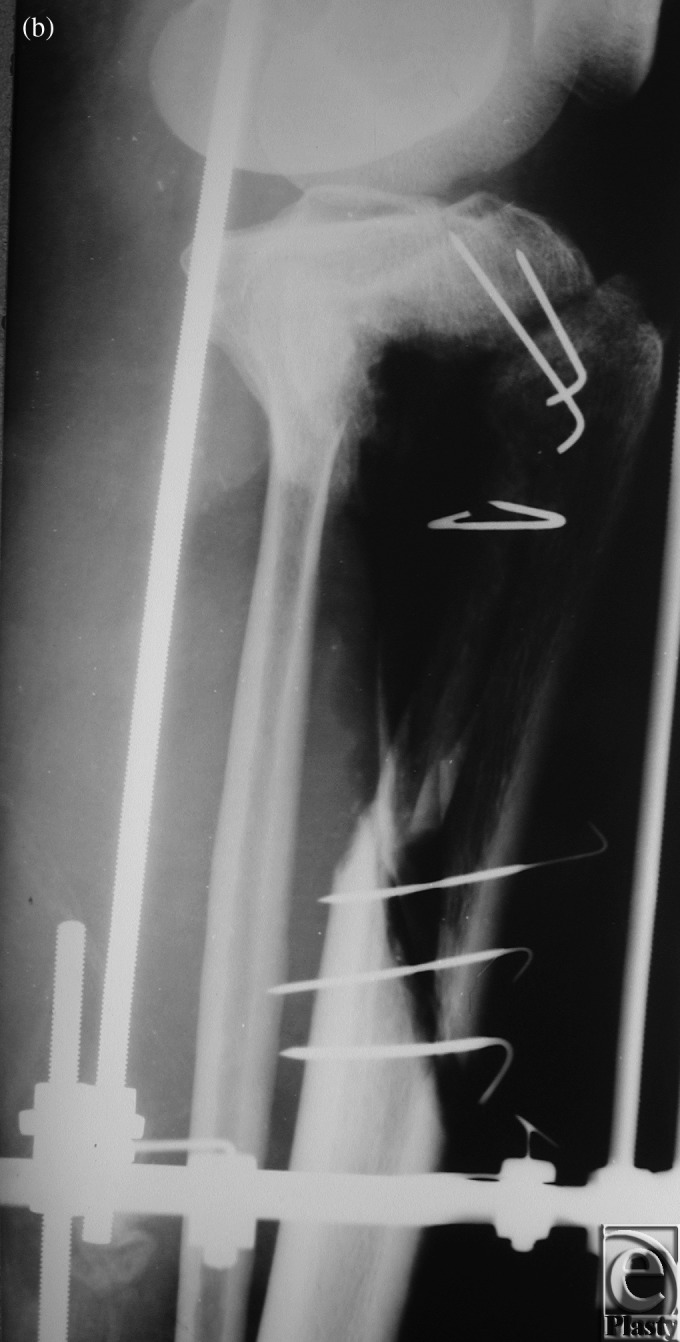

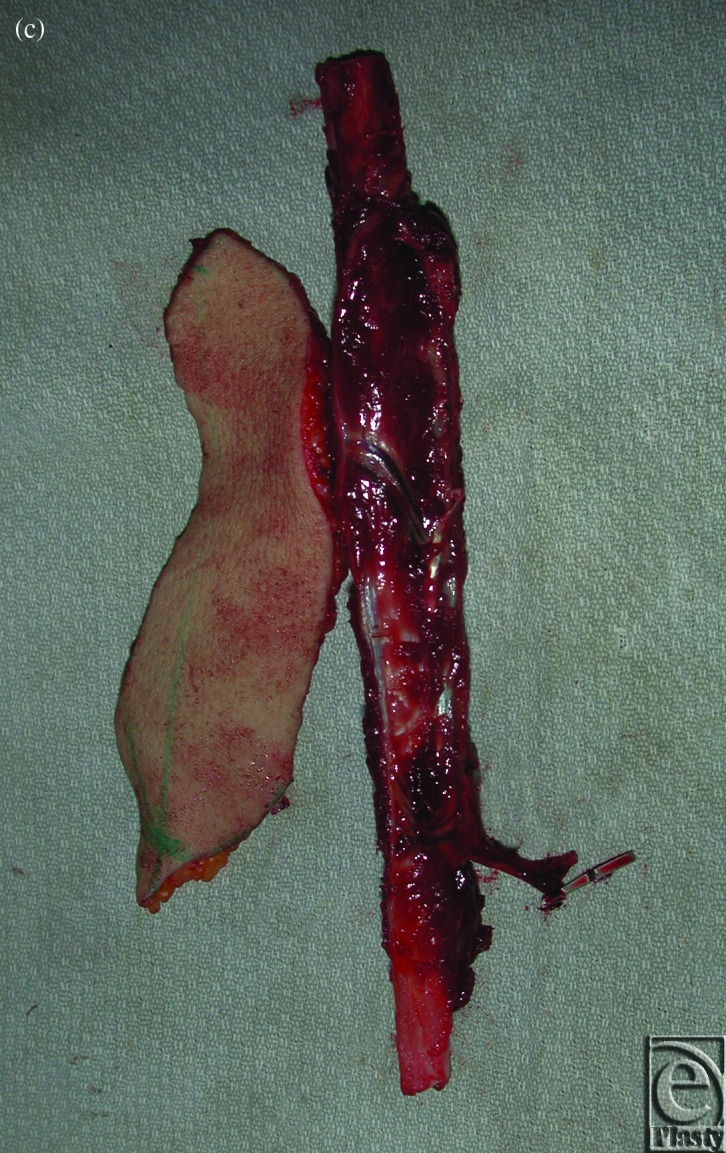

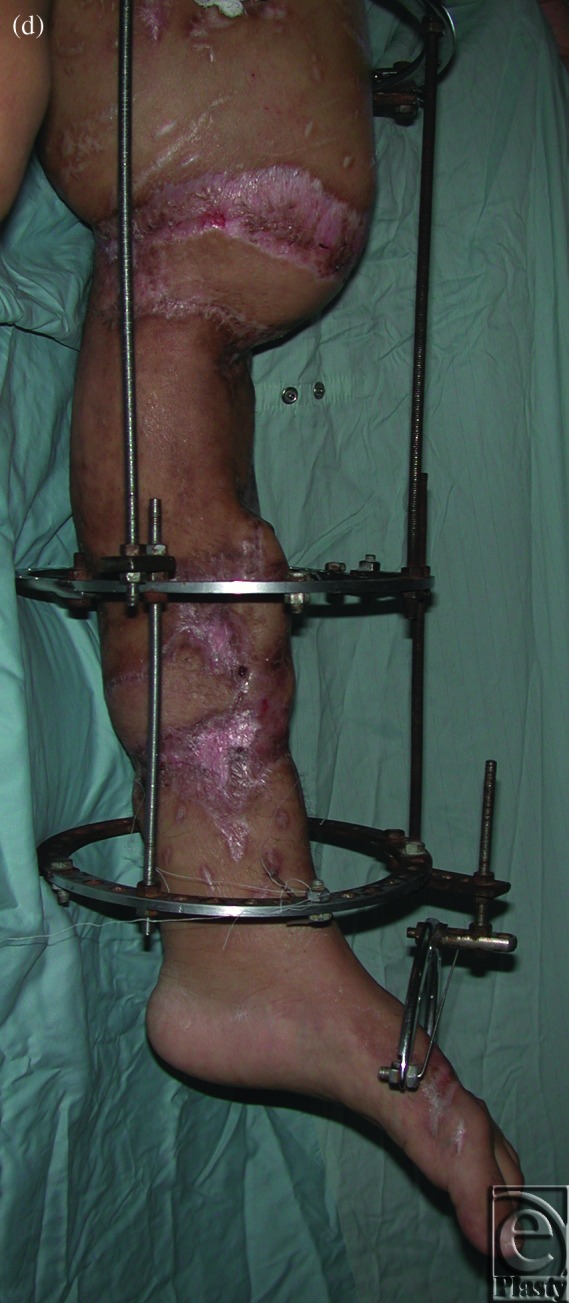

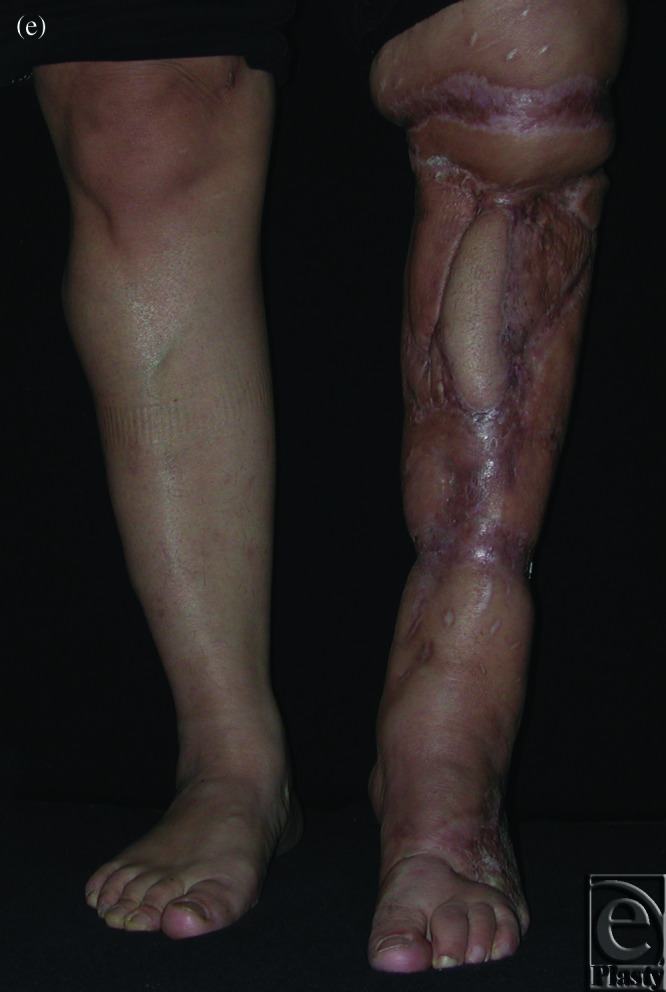
Case 2: Trauma of the lower leg. (*a*) Correction of the deformity by applying the Ilizarov device, (*b*) Preoperative x-ray, (*c*) Fibula osteocutaneous flap, (*d*) External fixation, (*e*) The 1-year postoperative view, and (*f*) A 1-year postoperative x-ray.

The patient was a 35-year-old woman who suffered from necrosis of both the tibia and fibula as a result of open trauma. Full debridement and temporary full-thickness skin grafting were performed in primary hospitals before we applied our 2-stage method. After correcting the deformities by applying the Ilizarov device in our hospital, an 18-cm fibula osteoseptocutaneous flap was found to be necessary and was transferred from the contralateral side. End-to-end anastomoses were performed between peroneal and posterior tibial vessels (Figs 2a-2f).

**Case 3: Osteomyelitis of the lower leg**

**Figure 3:**
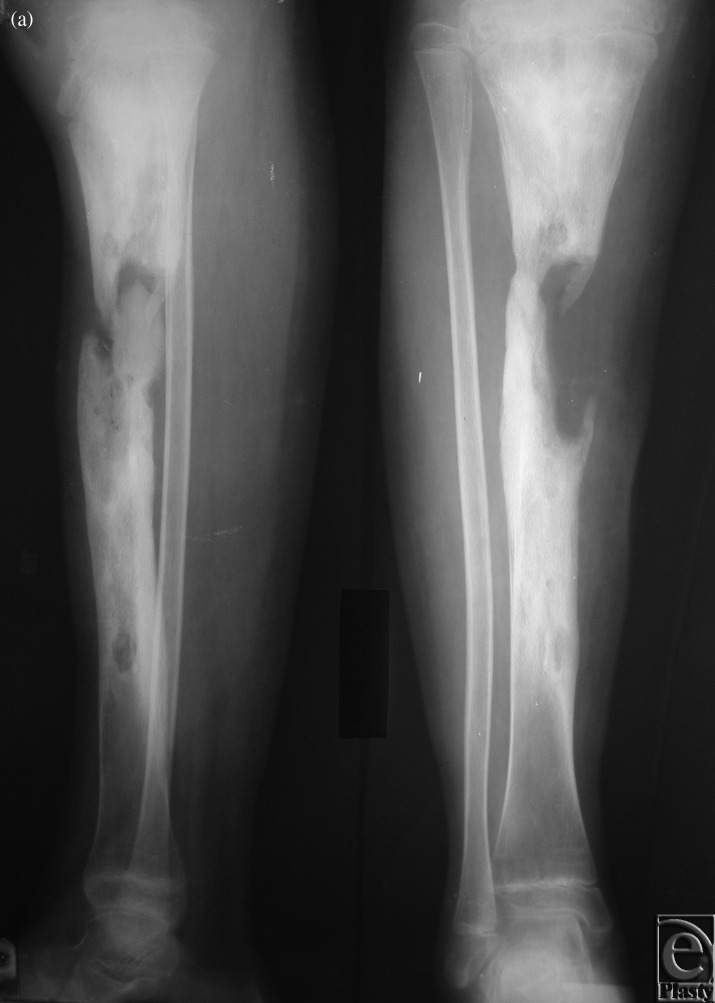

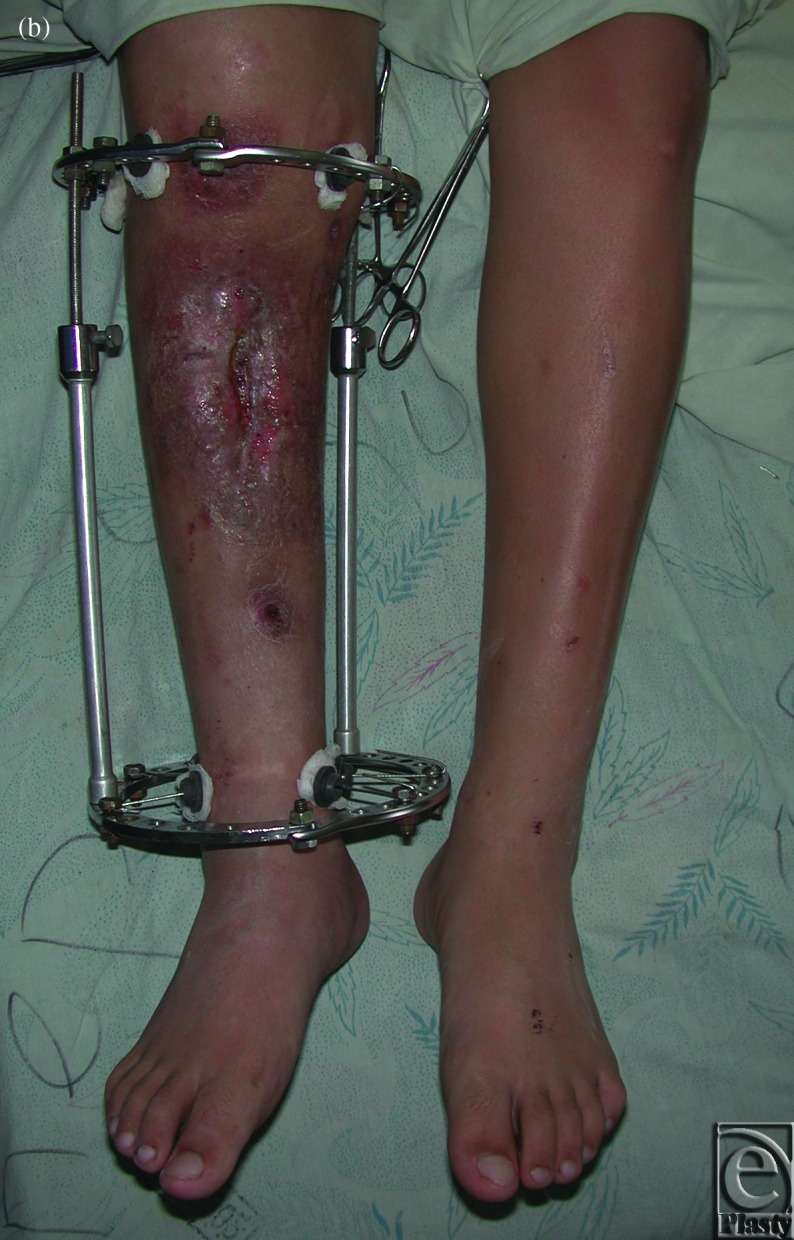

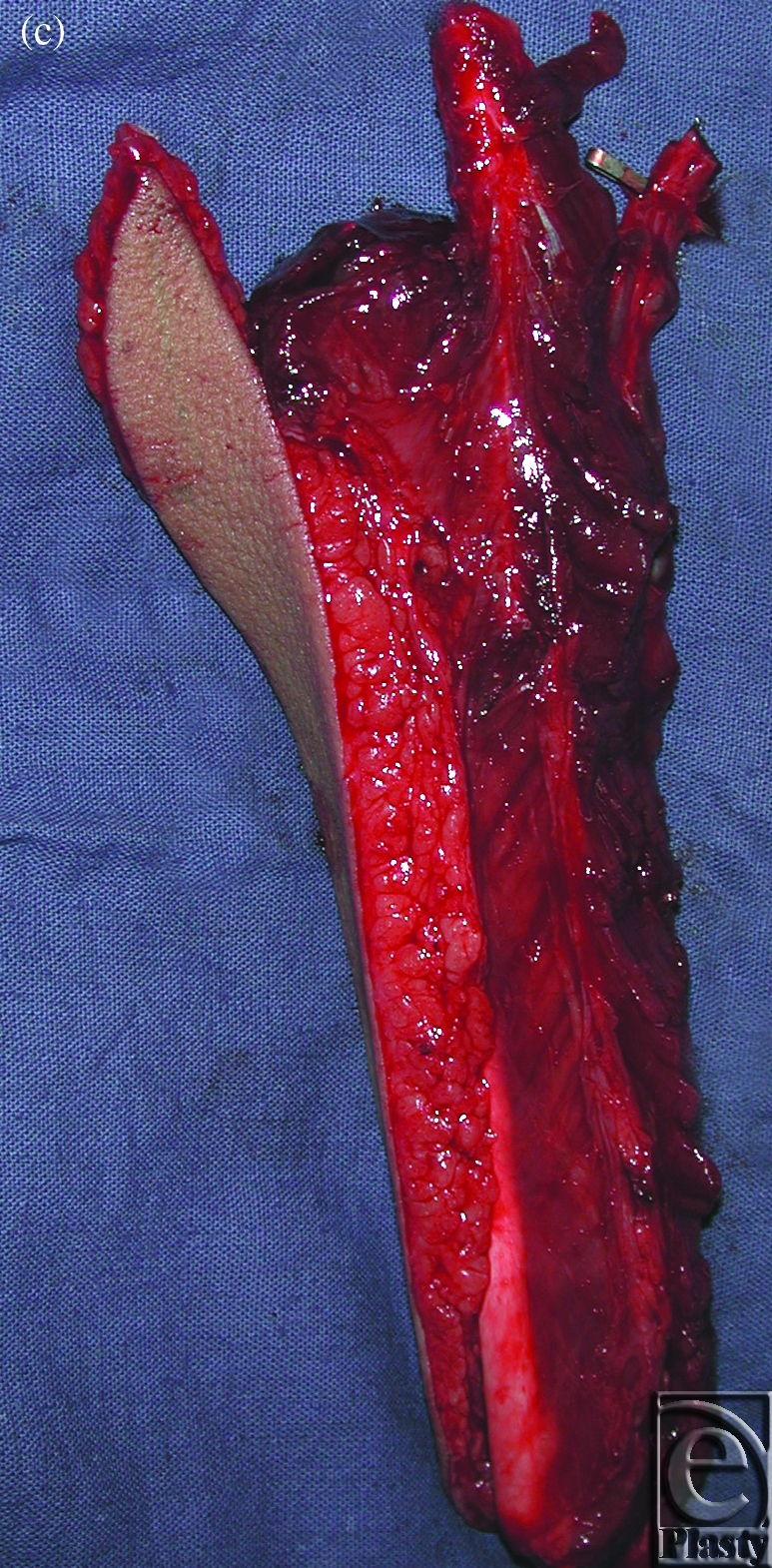

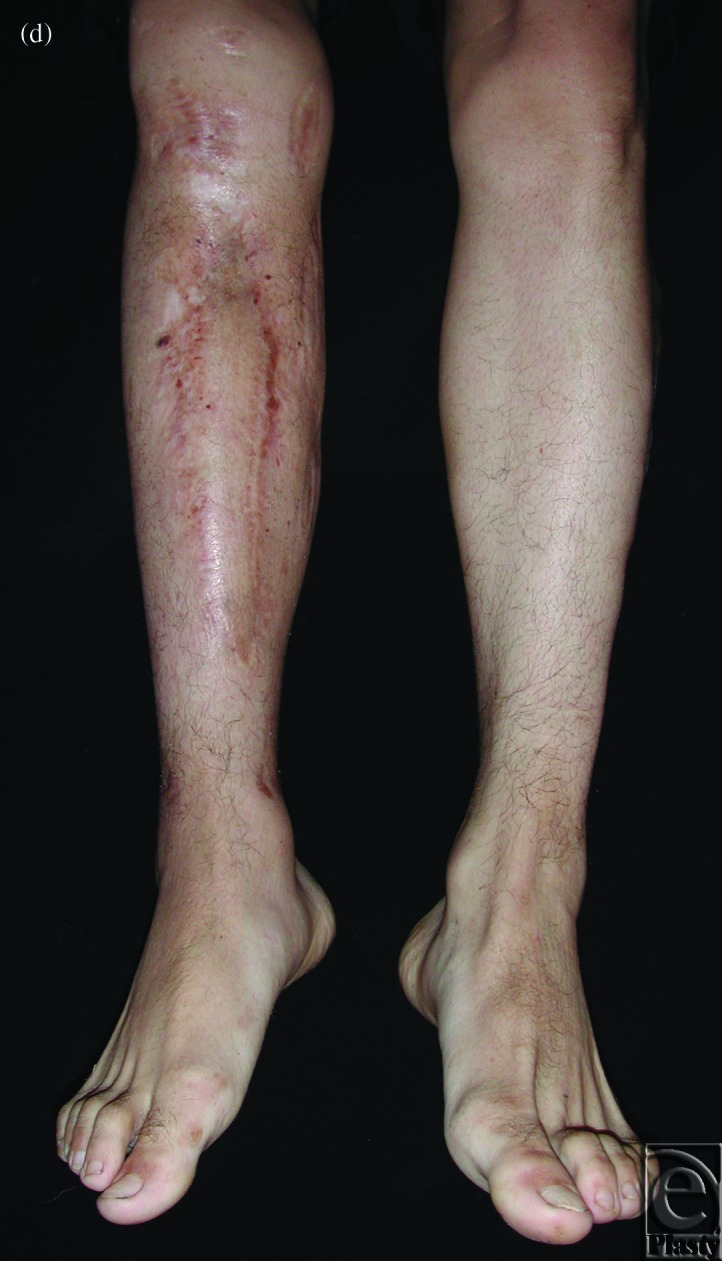

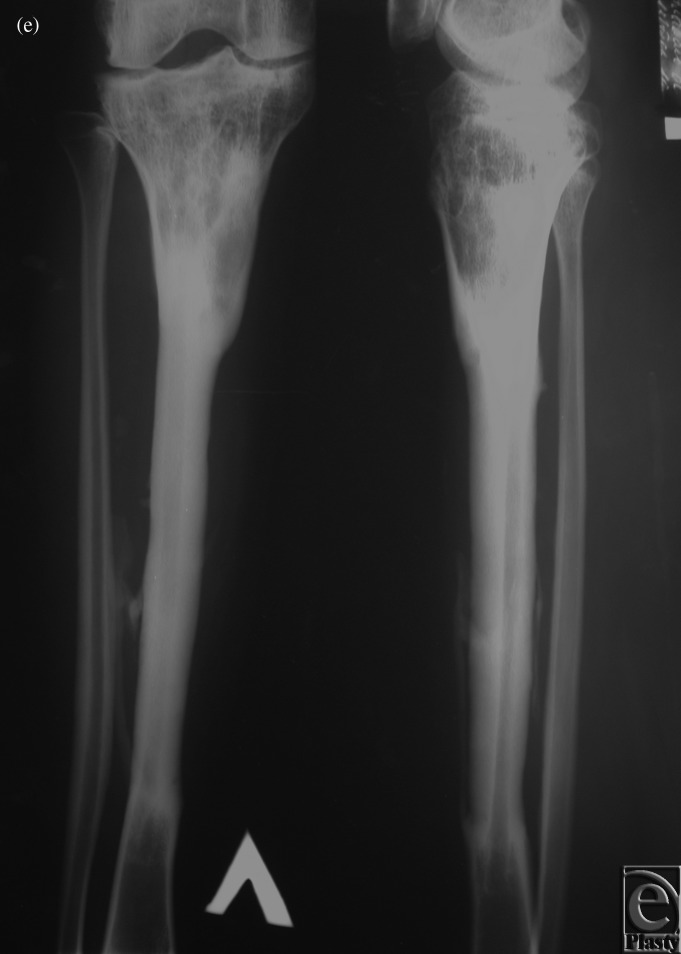
Case 3: Osteomyelitis of the lower leg. (*a*) Preoperative x-ray, (*b*) External fixation, (*c*) Fibula osteocutaneous flap, (*d*) The 2-year postoperative view, and (*e*) An x-ray 2 years after surgery.

The patient was a 28-year-old man who suffered from hematogenous osteomyelitis of the tibia. Before being transferred to our center, he underwent 4 separate debridements. After a 23-cm-long space between the 2 ends of the affected tibia was obtained by Ilizarov external fixation in our hospital, a free fibula osteoseptocutaneous flap was harvested and transferred to cover both the bone and the soft tissue defect. The external fixator was removed 8 months after the free flap transfer (Figs 3a-3e).

**Case 4: Tumor of the forearm**

**Figure 4:**
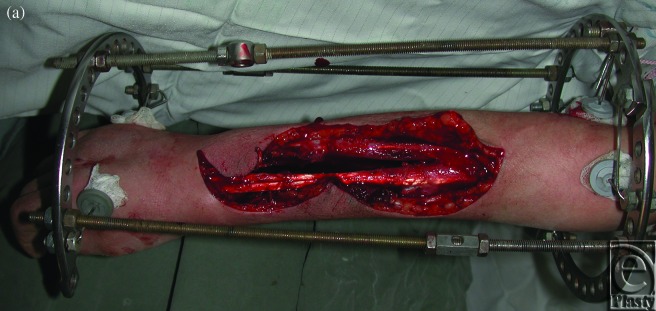

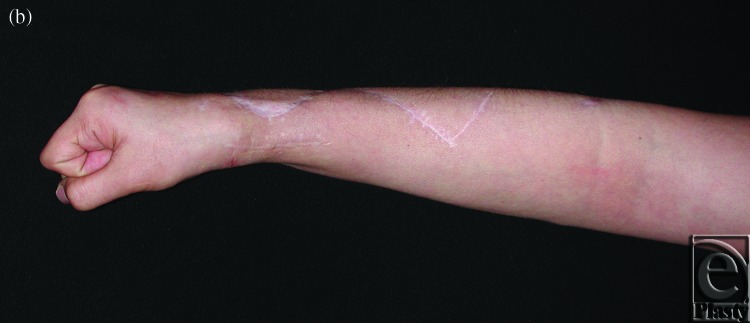

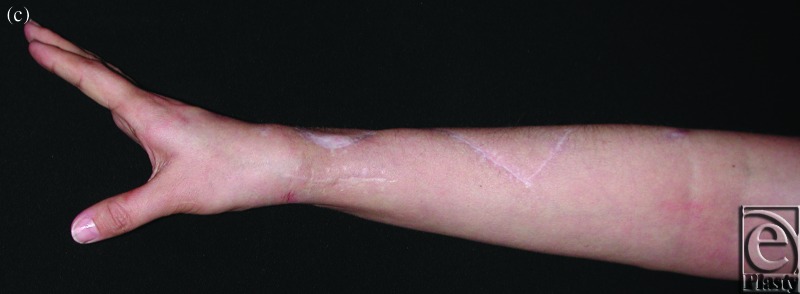

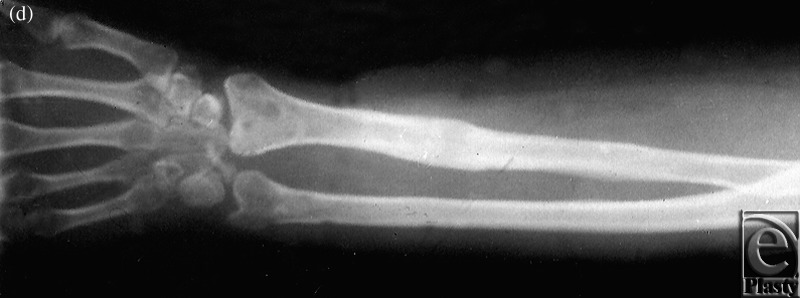
Case 4: Tumor. (*a*) Fibula osteocutaneous flap, (*b*) The 8-month postoperative view, (*c*) An 8-month postoperative x-ray, and (*d*) The 2-year postoperative view.

The patient was a 23-year-old woman who suffered from a giant cell tumor on the distal part of the radius. In one surgical procedure, wide tumor excision was performed and an epiphyseal fibular osteoseptocutaneous flap was transferred to construct the radiocarpal joint. Vascular anastomosis between the peroneal and radial vessels was performed. An articular capsule (lateral radial ligament) was reconstructed by using a fibular collateral ligament. The Ilizarov device was applied and fixed for 5 postoperative months (Figs 4a-4d).

## DISCUSSION

The biggest challenges that clinicians face when performing large area limb salvages relate to the tissue defect, namely how much should and can be excised, and how to cover and restore it. This surgical challenge entails difficult balancing acts between deformity repair and tissue reconstruction, soft tissue and bone reconstruction, and the simultaneous restoration of the physical, mechanical, and esthetic features of the affected limb. Moreover, these objectives must all be achieved within a limited perioperative period. In addition, limb salvage procedures should be applicable to all large defects of the limbs whether they are due to congenital pseudarthrosis, traumatic fractures, infectious osteomyelitis, or benign/malignant tumors. Given these considerations, we performed an initial successful trial of the 2-stage reconstruction procedure on patients with congenital pseudarthrosis.^3^ We described here the staged reconstruction method, where the application of the Ilizarov device is combined with microsurgical osteoseptocutaneous transplantation, which we believe is the solution to the challenges posed by large area limb defects.

In the face of the wide resections needed as a result of congenital abnormalities, traumatic injury, inflammation, and tumors, the Ilizarov device application acts to repair while the free osteoseptocutaneous flap transplantation serves to reconstruct; in this fashion, these 2 components match each other well in the treatment of large area limb defects. In the 1-stage method, the Ilizarov device fixes both ends of the bone, allowing full resection of the pathological bone by an orthopedic specialist; this serves to prepare a *physically* safe bed for the primary free fibula transplantation while keeping the size and shape of the affected bone similar to that of the healthy bone on the contralateral side. In the 2-stage method, the Ilizarov device corrects the axial, angular, and translational deformities of the affected bones by serving as a dynamic fixator and tractor; this results in a *mechanically* safe bed. Thereafter, the only remaining problem is the tissue defect, which is repaired by the secondary free fibula transplantation performed by a plastic specialist.

This technique provides not only a safe bed due to the Ilizarov device, it also ensures that the transplanted bone in the recipient site is well-nourished and fully covered by healthy soft tissue; this is an essential requirement for the survival of such combined transplants.^4^ Thus, the use of the Ilizarov allows full resections of ischemic, infected, damaged, or scarred soft tissues as well as pathological bones in the recipient site without creating any worries about pathological fractures. This, together with the primary or secondary healthy soft tissue coverage from the donor site, whose size and shape matches well to the underlying hard tissue, significantly reduces the risk that the transplanted bones will undergo devascularization, sequestration, and consequent segmental bone necrosis.^5^

Apart from guaranteeing the physical continuity of the bone and its similarity in size and shape to that of the healthy contralateral bone, the staged reconstruction also maximizes mechanical restoration. The patient can be ambulatory and bear weight during fixation and dynamic traction with the Ilizarov device^5^ from a few days after microsurgery (the delay is needed to ensure the safety of the free flap). Thus, it is possible for the patient to engage in early activities and rehabilitations that reduce the risk of soft tissue dystrophy, cartilage malnutrition, and bone consolidation, which are often seen in procedures that employ long duration traditional external fixation.^6^ In addition, the Ilizarov provides dynamic and flexible fixation at no cost of accuracy that allows further postoperative adjustments. This means that bone-wedge removal, which can aggravate the shortage of bone,^6^ is not necessary. In contrast, bone-wedge removal is required in traditional internal fixation. In addition, the thin K-wire in the Ilizarov device prevents secondary injury to the adjacent tissues and potential growth disturbance of the bone. This is particularly important in pediatric cases.

In summary, we introduced here a staged approach where external fixation *via* the Ilizarov device is combined with microsurgical osteoseptocutaneous flap transplantation; this approach is suitable for large area limb salvages that result from congenital abnormalities, traumatic injury, inflammation/infection, and tumors. This staged approach seamlessly repairs and reconstructs the bone in combination with soft tissue reconstruction, thereby simultaneously restoring the functions and esthetics of the affected limb; this is all achieved with minimal costs in terms of time, money, and patient pain. We recommend others to try it.

## Figures and Tables

**Table 1:** Complications associated with staged reconstruction of large area limb defects

		Pseudarthrosis		Fracture		Osteomyelitis		Tumor	
		Congenital	Traumatic	Open	Close	Hematogeneous	Pyogenic	Giant cell	Osteosarcoma
Subtype		24	3	11	2	7	4	4	4
Location	Tibia	15	2	6	1	7	2	0	2
	Forearm	12	1	4	1	0	2	3	1
	Shoulder	0	0	1	0	0	0	1	1
	Vascular thrombosis (Artery)	0		0		2			0
	Vascular thrombosis (Vein)	0		1		0			0
Complication	Infection	0		0		2			0
	Nonunion	2		0		0			0
	Fracture	3		0		0			0
